# Colistin Dosage without Loading Dose Is Efficacious when Treating Carbapenem-Resistant *Acinetobacter baumannii* Ventilator-Associated Pneumonia Caused by Strains with High Susceptibility to Colistin

**DOI:** 10.1371/journal.pone.0168468

**Published:** 2016-12-19

**Authors:** Rocío Álvarez-Marín, Rafael López-Rojas, Juan Antonio Márquez, María José Gómez, José Molina, José Miguel Cisneros, Carlos Ortiz-Leyba, Javier Aznar, José Garnacho-Montero, Jerónimo Pachón

**Affiliations:** 1 Clinical Unit of Infectious Diseases, Microbiology and Preventive Medicine Infectious Diseases Research Group Institute of Biomedicine of Seville (IBiS) University of Seville/CSIC/University Hospitals Virgen del Rocio and Virgen Macarena, Seville, Spain; 2 Clinical Unit of Critical Care and Emergencies, Institute of Biomedicine of Seville (IBiS), University Hospital Virgen del Rocío/CSIC/University of Seville, Seville, Spain; 3 Clinical Unit of Critical Care, University Hospital Virgen Macarena, Seville, Spain; University of Colorado Denver, UNITED STATES

## Abstract

**Objectives:**

This study aims to analyze the mortality and the length of ICU stay (LOS) of *A*. *baumannii* VAP compared to respiratory colonization in patients with mechanical ventilation (MV).

**Methods:**

A prospective cohort study was performed in an ICU of adult patients (February 2010–June 2011). One hundred patients on MV with *A*. *baumannii* in lower respiratory airways were recruited, and classified as VAP or airways colonization according to CPIS criteria, with a punctuation ≥6. LOS, 30-days mortality, *A*. *baumannii* bacteremia, and clinical features including antibiotic therapy were recorded. Multivariate analysis (linear and Cox regression) and survival analysis (Kaplan-Meier curves) were performed.

**Results:**

Fifty-seven VAP and 43 colonized *A*. *baumannii* patients were analyzed. Among the *A*. *baumannii* strains, 99% were non-susceptible to carbapenems and the MIC_90_ of colistin was 0.12 mg/l. Therapy was appropriate in 94.6% of VAP patients, most of them with colistin 6 MIU/day, although in 13 (23.6%) cases colistin was started 48 hours after the onset of VAP. Mortality was similar in both groups (VAP 24.6% vs. colonized 27.9%, p = 0.7). Bacteremia and acute kidney insufficiency were associated with decreased survival (p = 0.02 and p = 0.04, respectively) in VAP patients. LOS was 21.5 (11.5–42.75) vs. 9 (6–22) days for VAP and colonized patients (p = 0.004). VAP (p = 0.003) and age (p = 0.01) were independently related to a longer LOS.

**Conclusions:**

Multidrug-resistant *A*. *baumannii* VAP treated with colistin does not have a different mortality compared to lower airways colonization, among patients on mechanical-ventilation, in a setting of high susceptibility to colistin of *A*. *baumannii*.

## Introduction

*Acinetobacter baumannii* is among the leading etiologies of hospital-acquired infections worldwide, ventilator-associated pneumonia (VAP) being the most common among them [1]. Attributable mortality of *A*. *baumannii* infections can be heterogeneous, depending on underlying conditions and appropriateness of antibiotic therapy, but it may have been as high as 28.5–44.5% [2].The studies that have assessed the attributable mortality of *A*. *baumannii* VAP usually included either patients with VAP caused by other pathogens or patients without any pulmonary infection [2–3] in the control group. Since the risk factors for acquisition of *A*. *baumannii* can be also associated to higher mortality (wide-spectrum antibiotic therapy, invasive devices and procedures, clinical severity, duration of the ICU stay) [4–5], attributable mortality of *A*. *baumannii* could be overestimated despite the efforts made to avoid confusing factors. Thus, mechanically ventilated patients with lower airways colonization by *A*. *baumannii* may be the most appropriate control group to weigh up the clinical impact of the *A*. *baumannii* VAP, considering that around half of all the patients acquiring *A*. *baumannii* in the respiratory tract develop VAP, while the others remain just colonized [6],and that risk factors for acquisition of *A*. *baumannii* are similar for both groups [5].

The spread of multidrug-resistant (MDR) strains, most of them resistant to carbapenems, has greatly complicated the management of *A*. *baumannii* infections. Colistin, which conserves activity against most clinical isolates of MDR *A*. *baumannii*, has emerged as its first-line therapy. However, the traditional dosing regimen of colistin (6million of international units [MIU] daily, tid) was considered insufficient to treat susceptible pathogens with a minimal inhibitory concentration in the upper limit of susceptibility, according to the results of multiple pharmacokinetics studies [7–8]. Consequently, higher dosages of colistin have been generally adopted, although a definitive consensus is lacking [9] and a loading dose is recommended to achieve early effective concentrations in critically ill patients [10]. In our center, where a predominance of carbapenem-resistant *A*. *baumannii* has been observed since 2008, the traditional dosages were used until 2011, when a new protocol was slowly adopted (loading dose of 4.5–6 MIU and 9 MIU daily, bid or tid).

The aims of this study were: i) To analyze the impact on mortality and ICU length of stay (LOS) of *A*. *baumannii* VAP compared to lower airways colonization in mechanically ventilated patients; ii) To evaluate the efficacy of the different dosages of colistin in *A*. *baumannii* VAP. We also analyzed the resistance mechanisms for carbapenems in the resistant *A*. *baumannii* isolates.

## Patients and Methods

### Design

This was a prospective, observational cohort study of patients on invasive mechanical ventilation. The study was approved by the Ethics Committee of the University Hospital Virgen del Rocío. Written informed consent was obtained from a relative of all patients before inclusion in the study.

### Setting and period

The study was conducted at the University Hospital Virgen del Rocío, a tertiary-care hospital with 1,251 beds, including 62 adult ICU beds. The enrollment period was from February 2010 to June 2011.

### Criteria of inclusion and exclusion

Adult patients (≥18 years) admitted to the ICU and requiring invasive mechanical ventilation for more than 48 hours, and having at least one culture of trachea-bronchial aspirate (BAS) with *A*. *baumannii* isolation. Patients with history of previous endotracheal intubation in the preceding 365 days, tracheotomy or cystic fibrosis, were excluded.

### Recruitment and follow up

Daily, two of the researchers identified the new candidates among ICU patients with MV. After obtaining the informed consent, their baseline characteristics were collected in a standardized form and BAS cultures were performed every three days while intubated, up to 30 days. If *A*. *baumannii* was isolated, patients were definitively included in the study cohort and followed until hospital discharge, death or 30 days, which ever occurred first. All clinical decisions were made by the physicians in charge of the patients.

### Variables

Patient demographics, primary diagnosis, in-hospital admission department, APACHE II [11] and Charlson comorbidity Score [12] were recorded at the moment of inclusion. Antimicrobial therapy reception, and development of any cause of septic shock or acute kidney injury (AKI) were recorded if they happened at any time during the follow-up, as well as ICU LOS and mortality.

Patients were classified as *A*. *baumannii* VAP or lower airways colonization according to the CPIS score, being classified as VAP those cases with a punctuation ≥6 [13]. Septic shock [14] and AKI [15] were defined according to standard criteria. Appropriate antimicrobial therapy was considered when VAP patients received at least one drug active against the *A*. *baumannii* isolates. Mortality was defined as death from any cause within 30 days after the first *A*. *baumannii* isolation in colonized patients or after the onset of pneumonia in infected patients. ICU stay was measured from the first isolation of *A*. *baumannii*.

### Microbiological procedures

BAS were processed immediately for quantitative cultures. One hundred μL aliquots of serial ten-fold dilutions were plated on Columbia sheep blood agar and incubated at 37°C. Colony forming units (CFUs) were counted after 24 h and expressed as log_10_ CFU/mL. Blood cultures were processed in a BACTEC™ system. *A*. *baumannii* identification was performed by biochemical tests in an automatized multi-test system (MicroScan Walkaway™), and by matrix-assisted laser desorption ionization–time-of-flight (MALDI-TOF) analysis using a MALDI biotyper (Bruker Daltonics). Among isolates from BAS, the first one from colonized patients and the first at the diagnosis in patients with VAP were selected for antimicrobial susceptibility testing. Minimum inhibitory concentrations for imipenem, sulbactam, ceftazidime, amikacin, ciprofloxacin, tigecycline, colistin, and rifampin were performed by the broth microdilution according to the CLSI criteria [16]. Isolates were defined as multi-drug resistant (MDR) according to the standard criteria [17]. In those isolates resistant to imipenem, PCR amplifications were done to detect the presence of the oxacillinases*bla*OXA51, *bla*OXA23, *bla*OXA24, *bla*OXA58, and the metallo-β-lactamases (MBL) IMP and VIM genes [18–19]. Expression of *bla*OXA51 was assessed testing by PCR the presence of the sequence IS*Aba*1 upstream and the gene encoding *bla*OXA51 [20].

### Sample size

For the sample size, assuming 49,25% mortality in patients with *A*. *baumannii* VAP (average mortality of *A*. *baumannii* VAP in two studies performed in our centre [3,21]) and 20% in respiratory colonization [6], and an infected and colonized ratio of 1:1, a minimum of 40 evaluable patients per group were required, with an alpha error of 5% and a power of 80%.

### Statistical analysis

Discrete variables were expressed as counts (percentage) and continuous variables as the median and interquartile range or mean and standard deviation, as appropriate. ICU stay analysis was made only on patients who survived until discharge from the ICU. Logarithmic transformation was performed if length of stay variables did not show linear distribution. Differences in categorical variables were calculated using a two-sided likelihood ratio chi-square test or Fisher exact test, and the *t-*test or Mann-Whitney test was used for continuous variables, when appropriate. Survival was analyzed by Kaplan-Meier curves. Back stepped Cox regression was used to assess the factors independently associated to survival. Results are presented as the hazard ratio (HR) and 95% confidence interval (CI). Linear regression was used for multivariate analysis in the case of ICU LOS, including the variables with a p<0.05 in the bivariate analysis. Statistical significance was defined as p<0.05. The PASW 18.0 package was used.

## Results

**Fig 1:**
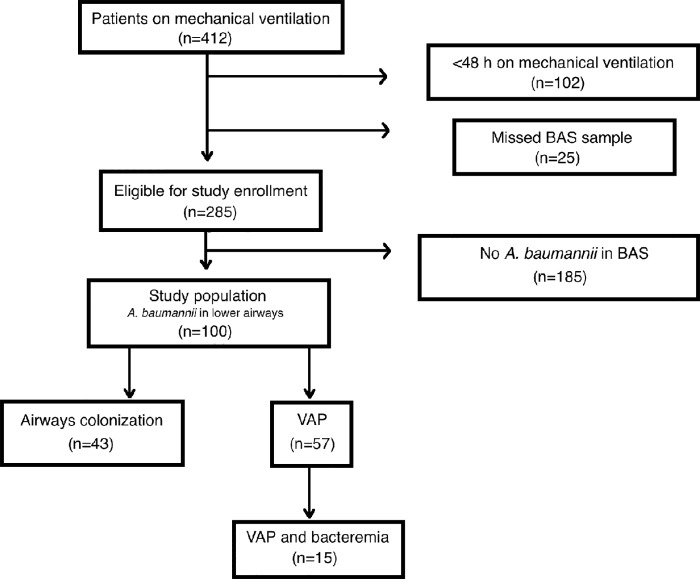
Flow chart on the enrollment of patients in the study and final classification as ventilator-associated pneumonia (VAP) or colonization in those with isolation of *Acinetobacter baumannii* in trachea-bronchial aspirate (BAS).

During the study period, 285 eligible patients were identified in the ICU. *A*. *baumannii* was isolated from respiratory samples in 100 of them: 57 were classified as VAP, with an average CPIS score of 6.36±1.14, and 43 as lower airways colonization, respectively (Fig 1). The elapsed time from intubation to the first isolate was 9 (5–14) days.

Colonized and VAP patients had similar demographic and clinical characteristics, as detailed in Table 1. Fifty-three (92.9%) infected patients received appropriate therapy with colistin and one (1.7%) with tigecycline; in 13 (23.6%) cases, colistin was started 48 hours after the onset of VAP. Among patients who received colistin, loading dose was not used and all of them received dosages of 2 MIU of sodium colistimethate thrice daily, titrated on renal function when appropriate.

**Table 1:** Distribution of baseline and clinical features among patients with *A*. *baumannii* lower airways colonization and those with *A*. *baumannii* VAP.

	Colonization	VAP	p
	(n = 43)	(n = 57)	
**Age**	66 (43–70)	51 (43.5–69.5)	0.186
med (IQR)			
**Female sex**	17 (39.5)	22 (38.6)	0.920
n (%)			
**Charlson score**	1 (0–2)	0 (0–2)	0.529
med (IQR)			
**Chronic heart failure**	7 (16.3)	6 (10.5)	0.397
n (%)			
**COPD**	5 (11.6)	6 (10.5)	0.862
n (%)			
**DM**	9 (20.9)	11 (19.3)	0.840
n (%)			
**AIDS**	0	1 (1.8)	0.383
n (%)			
**Chronic liver disease**	1 (2.3)	2 (3.5)	0.215
n (%)			
**Medical patient**	24 (52.2)	22 (38.6)	0.084
n (%)			
**Surgical patient**	8 (18.6)	17 (29.8)	0.200
n (%)			
**Trauma patient**	11 (25.6)	18 (31.6)	0.510
n (%)			
**APACHE II**	17 (12–21.25)	17 (13–20)	0.947
med (IQR)			
**Previous antibiotic therapy**	40 (93)	53 (92.9%)	0.994
n (%)			
**Carbapenems**	13 (30.2)	33 (57.9)	0.006
n (%)			
**Piperacillin-tazobactan**	10 (23.2)	25 (43.8)	0.032
n (%)			
**Quinolones**	14 (32.6)	14 (24.6)	0.378
n (%)			
**Vancomycin**	6 (13.9)	22 (38.6)	0.007
n (%)			
**Acute kidney injury**	13 (30.2)	27 (47.4)	0.085
n (%)			
**Septic shock**	17 (39.5)	30 (52.6)	0.194
n (%)			
**Surgery**	19 (44.2)	38 (66.7)	0.025
n (%)			
**Mortality**	12 (27.9)	14 (24.6)	0.700
n (%)			

**Fig 2:**
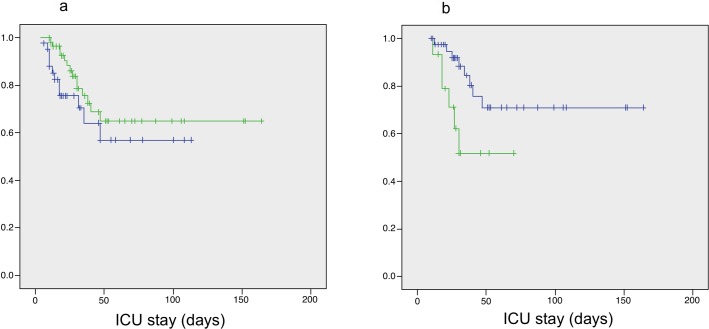
**a) Survival Kaplan-Meyer analysis between *Acinetobacter baumannii* colonized and ventilator-associated pneumonia (VAP) patients** (Blue: Colonized patients; Green: VAP patients); **b) Survival Kaplan-Meyer analysis among patients with *Acinetobacter baumannii* ventilator-associated pneumonia, with or without bacteremia.** (Blue: non-bacteremic VAP patients; Green: bacteremic VAP patients).

There were no differences in the mortality of patients with *A*. *baumannii* VAP regarding to those with airways colonization (Table 1), and so did their survival rate according to the Kaplan-Meier analysis (p = 0.3) (Fig 2A). Patients with bacteremic VAP had a trend to higher mortality than VAP patients without bacteremia (42.9% *vs*. 20.9%, p = 0.1); and the survival Kaplan-Meier analysis showed lower survival in bacteremic patients (p = 0.02) (Fig 2B). In the multivariate survival analysis, colistin was associated to a better survival in patients with VAP, while bacteremia and AKI were related to poor prognosis (Table 2).

**Table 2:** Bivariate and multivariate survival analysis for mortality in patients with *A*. *baumannii* ventilator-associated pneumonia.

Variable	Dead	Survivors	Bivariate		Multivariate	
	(n = 14)	(n = 43)	HR (95%CI)	p	HR (95%CI)	p
**Female sex**	7 (50)	15 (34.9)	1.22 (0.42–3.48)	0.710		
n (%)						
**Age**	58 (43.25–76.75)	49 (43–65)	1.01 (0.97–1.04)	0.580		
med (IQR)						
**Charlson Score**	2 (0.75–2.25)	0 (0–1)	1.27 (0.95–1.7)	0.107		
med (IQR)						
**APACHE II**	19.5 (17.75–20.25)	15.5 (12.25–19.5)	1.01 (0.95–1.08)	0.632		
med (IQR)						
**Medical patient**	8 (57.1)	14 (32.6)	2.27 (0.79–6.57)	0.129		
n (%)						
**Surgical patient**	5 (35.7)	12 (27.9)	1.38 (0.46–4.13)	0.563		
n (%)						
**Trauma patient**	1 (7.1)	17 (39.5)	0.15 (0.02–1.12)	0.064		
n (%)						
**Septic shock**	11 (78.6)	19 (44.2)	2.26 (0.63–8.13)	0.212		
n (%)						
**Acute kidney injury**	11 (78.6)	16 (37.2)	3.5 (0.97–12.54)	0.055	**3.88 (1.06–14.21)**	**0.04**
n (%)						
**Colistin**	13 (92.9)	40 (93.0)	0.03 (0.002–0.47)	0.013	**0.02 (0.001–0.72)**	**0.033**
n (%)						
**Bacteremia**	6 (42.9)	9 (20.9)	4.49 (1.52–13.3)	0.007	**4.67 (1.53–14.25)**	**0.02**
n (%)						

Median ICU LOS was 17 days (8–33) for the whole sample, and 9 (6–22) *vs*. 21.5 (11.5–42.75) days for colonized and VAP patients, respectively (p = 0.004). Development of *A*. *baumannii* VAP (p = 0.003) and elder age (p = 0.013) were independently associated to a longer ICU stay (Table 3).

**Table 3:** Factors associated to the length of intensive care unit-stay (bivariate and multivariate analysis) in the whole cohort (n = 100).

Variable	Bivariate analysis		Multivariate analysis	
	p	OR (95%CI)	p	OR (95%CI)
**VAP** *	0.007	1.81 (1.18–2.79)	0.004	1.88 (1.23–2.88)
**Age**	0.041	1.01 (1.00–1.03)	0.010	1.01 (1.00–1.03)
Female sex	0.097	1.45 (0.93–2.26)	-	-
Charlson Score	0.257	1.45 (0.90–1.44)	-	-
APACHE II	0.03	1.04 (1.00–1.07)	0.47	1.01 (0.98–1.05)
Septic shock	0.011	1.80 (1.15–2.81)	0.204	1.35 (0.85–2.15)
Acute kidney injury	0.015	1.84 (1.13–3.01)	0.107	1.5 (0.91–2.47)
Bacteremia	0.98	0.99 (0.50–1.97)	-	-

* VAP: ventilator-associated pneumonia

Antimicrobial susceptibility is detailed in Table 4. Just one isolate was susceptible to imipenem. The MIC_90_ of colistin was 0.12mg/L. Ninety (90%) isolates were MDR.*bla*OXA51, which is constitutive in *A*. *baumannii*, was present in all the isolates, but just 9 of them presented the upstream sequence IS*Aba-*1. *bla*OXA58 was present in 81 isolates and *bla*OXA40 in 18 isolates, with an imipenem MIC_50_ of 16 and 64 mg/L, respectively (p<0.001); none of them were MBL producers. There were no differences in antimicrobial susceptibility/resistance between isolates from colonized and VAP patients.

**Table 4:** Antimicrobial susceptibility and mechanism of resistance to carbapenems in the isolates of *A*. *baumannii*.

Antimicrobial agent	MIC range	MIC_50_	MIC_90_	% susceptible isolates
**Imipenem**	0.25–256	16	128	1
**Meropenem**	0.38->8	>8	>8	1
**Amikacin**	0.25–64	8	32	78
**Ceftazidime**	16–256	64	128	0
**Ciprofloxacin**	0.5	32	128	0
**Colistin**	0.06–0.12	0.06	0.12	100
**Rifampicin**	1–128	32	128	15
**Sulbactam**	2–32	4	16	62
**Tigecycline**	0.06–0.5	0.25	0.25	100
	**n**		**Imipenem MIC**_**50**_	
***bla*OXA-58**	81		16	
***bla*OXA-40**	18		128	
**IS*Aba-1*-*bla*OXA51**	9		128	
**IMP**	0		-	
**VIM**	0		-	

## Discussion

In this prospective cohort, *A*. *baumannii* VAP did not produce an excess of mortality with respect to the lower airways colonization by the same pathogen in critically ill patients on MV. Nonetheless, VAP patients had a longer ICU stay, which points out the morbidity that it causes among patients on mechanical ventilation. Colistin at traditional low doses was used in almost all the patients with VAP; the high susceptibility to this drug among the *A*. *baumannii* isolates (MIC_90_ = 0.12 mg/L) could explain the lack of differences in mortality. Inside the group of patients with VAP, bacteremia had a poorer survival rate (p = 0.02). Given that the impact on mortality of VAP is related to its severity [21], the presence of concomitant *A*. *baumannii* bacteremia, whose crude mortality in the setting of pneumonia is high [22], can be understood as a key event that warns on severity and invasiveness of the VAP. A previous surgical procedure was more frequent in VAP than in colonized patients, but this feature was not associated to mortality in the whole sample (data not shown).

The crude mortality described for any-cause VAP is usually higher, ranging between 34.5% and 84% [3,23–25]. In this context, *A*. *baumannii* VAP has been associated to higher mortality and longer LOS in ICU than VAP caused by other pathogens [21, 26–28]. For instance, in a cohort of 163 patients with VAP, *A*. *baumannii* was identified as an independent risk factor for fatal outcome (OR 3.3, CI 1.12–9.7), reaching a crude mortality of 61% [26]. Carbapenem-resistance in *A*. *baumannii* has been associated to increased VAP mortality. A matched case-control study comparing 60 patients with *A*. *baumannii* VAP and 60 controls (patients with VAP caused by other microorganisms and patients without VAP), did not report differences in mortality for the whole sample [3]. Nevertheless, the sub-analysis of the patients with VAP by imipenem-resistant *A*. *baumannii* showed differences in mortality between cases and their matched controls (44% *vs*. 24%). In the present study, despite having found almost a 100% of carbapenem-resistance, mortality was quite lower than that previously described for *A*. *baumannii* VAP.

This discrepancy in the mortality could be explained by an unequal distribution in the appropriateness of antibiotic therapy among different studies. A meta-analysis on 24,186 patients from 44 observational studies [28] found that VAP was associated with higher mortality (OR 1.96, 95%CI 1.26–3.04) than their comparative groups; notwithstanding, this association was not found in VAP patients with appropriate initial treatment. The higher mortality of VAP caused by more resistant strains is probably related to the less likelihood of receiving appropriate therapy [21]. Therefore, despite the high level of resistance of the isolates included in this study, the rate of VAP patients receiving appropriate treatment (≈95%) has probably contributed to a lower mortality.

It is noteworthy that the antibiotic used for most patients in the present study was colistin, which was selected as an independent predictor of better survival. Until it became almost the only alternative, colistin was considered a last option for the treatment of *A*. *baumannii* infections due to concerns about its efficacy and safety. In a systematic review including all the studies assessing the efficacy of colistin compared to other antibiotics, the global analysis favored the comparators [29]. However, several pharmacokinetics studies have prompted that this lack of efficacy would be related to an insufficient antibiotic exposure, and higher doses have been proposed to obtain an optimal drug concentration at the infection site [7–8]. Dose-fractionation studies of colistin against *A*. *baumannii* in mouse infection models have revealed that AUC/MIC correlates with bacterial killing *in vivo* [30], even though its optimal value has not been well defined yet. In an *A*. *baumannii* murine pneumonia model, an AUC_0-24_/MIC of 158.8 was associated to a 3.78-log bacterial kill target in lungs and a reduction of 40% in mortality [31]. Karnik *et al*. [32] performed a PK study in patients with VAP caused by multidrug-resistant bacteria treated with 2 MU every 8 hours of colistin, the same kind of patients and colistin dosage as used in the present study, achieving an AUC_0-24_ of colistin of 47.1 mg^.^h/l. Taking into account the colistin MIC_90_ of 0.12 mg/L for the *A*. *baumannii* strains causing VAP in the present study, we would have achieved an average AUC_0-24_/MIC of 392.5 from the first colistin dose. Therefore, the low MIC_90_ of the included strains may explain the high effectiveness of the colistin therapy in this cohort of patients.

There is little further information on the association between MIC of colistin and effectiveness, because most clinical studies do not provide the MIC of colistin against the included strains. Markou *et al*. [33], in an observational study of 10 patients with *A*. *baumannii* infections, found that the MIC_90_ of colistin was higher among patients without clinical response to this drug (1 *vs*. 0.5 mg/L); however, the small sample size and the likely confounding factors preclude from assuming the impact of the MIC on the outcome. Recently, Kim *et al*. [34] reported a similar efficacy of tigecycline and colistin in a cohort of 70 patients with *A*. *baumannii* VAP. In this study, low tigecycline MIC was suggested as the reason for the good successful rate and survival among patients treated with tigecycline; however, unfortunately, the information regarding the precise colistin and tigecycline MIC was not reported.

In recent years, all efforts for the optimization of the treatment with colistin have been aimed at enhancing its pharmacokinetics, targeting to obtain higher levels, although safety can be of concern [35–36]. Focusing on strategies addressed to pharmacodynamics parameters related to the colistin MIC_90_ against the *A*. *baumannii* strains in each hospital might help to avoid over-dosage in some patients at risk of toxicity. With this strategy, the main concern would be if low dosages might select more easily resistant strains. However, this drawback seems to be ruled out by recent *in vitro* models, in which the emergence of resistant strains was paradoxically increased with higher colistin concentrations [37–38].

Our study has several limitations. First of all, the sample size was calculated a priori and the observed mortality of VAP patients was lower than expected. This made our sample size sub-optimal for detection of differences in mortality. Second, the method chosen for enrolling patients, periodic quantitative BAS cultures, has probably prompted the early initiation of appropriate antimicrobial therapy and influenced VAP outcome. Third, there is not a gold standard to classify patients as suffering from VAP and the use of CPIS score and other criteria, beyond the clinical ones, is a matter of debate; in fact, in the recent IDSA/ATS Guidelines for the Management of Adults With Hospital-acquired and Ventilator-associated Pneumonia [39] the authors do not recommend any criteria, among those reviewed, to support or exclude the HAP/VAP, because of low sensitivity and specificity. However, to avoid the selection bias of the research it is necessary to use specific criteria, in spite of the problems with the sensitivity/specificity; in this context, we decided to use the CPIS score, because it includes the clinical criteria (fever, purulent sputum, new infiltrates in the X-ray, respiratory insufficiency, and leukocytosis), with different punctuation to compose the reached score value. Fourth, the possibility of ventilator-associated trachea-bronchitis or airways infection produced by other microorganisms, or the occurrence of different events including other infections after the recovery or during the ICU stay that may have influenced individual outcomes, as is common in critically ill patients, were not considered.

In summary, the present study shows that MDR*A*. *baumannii* VAP treated with colistin does not have different mortality compared to lower airways colonization among patients on mechanical-ventilation. The high susceptibility to colistin among the isolated strains probably determined this similar outcome. In our opinion, this fact deserves special attention in relationship to the strategy in reaching the most appropriate treatment. Finally, bacteremic VAP showed a decreased survival in comparison to non-bacteremic VAP.
